# The anti-phytoalexin gene *Bx-cathepsin W* supports the survival of *Bursaphelenchus xylophilus* under *Pinus massoniana* phytoalexin stress

**DOI:** 10.1186/s12864-019-6167-2

**Published:** 2019-10-26

**Authors:** Feng Wang, Qiaoli Chen, Ruizhi Zhang, Danlei Li, Yaming Ling, Ruiqing Song

**Affiliations:** Key Laboratory of Sustainable Forest Ecosystem Management-Ministry of Education, School of Forestry, Northeast Forestry University, Harbin, 150040 Heilongjiang People’s Republic of China

**Keywords:** Phytoalexin, Carvone, *Bursaphelenchus xylophilus*, *Bx-cathepsin W*, WGCNA

## Abstract

**Background:**

Pine trees challenged by *Bursaphelenchus xylophilus* invasion produce phytoalexins to combat this nematode. Nevertheless, the phytoalexins of Asian pine trees are ineffective against *B. xylophilus*. The anti-phytoalexin genes of *B. xylophilus* disable almost all Asian pine phytoalexins, which has allowed *B. xylophilus* to devastate pine forests in eastern Asia over the last four decades. However, to date, the factors that stimulate anti-phytoalexin gene expression and the mechanisms by which these genes act are not well understood.

**Results:**

Here, we described anti-phytoalexin genes in *B. xylophilus* using transcriptomic and bioinformatics analyses. The genes that were induced by both *Pinus massoniana* and carvone and had similarly elevated expression trends were considered anti-phytoalexin genes. Altogether, 187 anti-phytoalexin genes were identified, including 4 cathepsin genes. KEGG pathway enrichment indicated that those cathepsins were related to the Lysosome pathway. Since cathepsins help to maintain metabolic homeostasis by participating in the degradation of heterophagic and autophagic material, the lysosomal cathepsin gene *Bx-cathepsin W* was cloned and characterized. The results of the RNAi assessment indicated that the knockdown of *Bx-cathepsin W* reduced the survival rates of *B. xylophilus* under carvone or *P. massoniana* stress. The correlation between *Bx-cathepsin W* and the susceptibility of pines showed that *Bx-cathepsin W* might help improve the anti-phytotoxin ability of *B. xylophilus*.

**Conclusions:**

The results indicated that the anti-phytoalexin gene *Bx-cathepsin W* supported the survival of *B. xylophilus* under *P. massoniana* phytoalexin stress. The cDNA library sequencing, differentially expressed gene identification, and WGCNA algorithm analysis provided insight at a systemic level into the gene regulation of *B. xylophilus* in response to the immune reaction of *P. massoniana*. These results will lead to a better understanding of the function of nematode defenses in host innate immunity.

## Background

The pine wood nematode *Bursaphelenchus xylophilus*, the causative agent of a devastating epidemic of pine wilt disease, is a migratory endoparasite that causes severe damage to pine forests in East Asia and Europe [1, 2]. In both the nematode’s native habitat in North America and in new epidemic areas in East Asia and Europe, *B. xylophilus* has evolved tolerance to pine defenses. Especially in China [3] and Japan, the nematode can completely defuse all natural host resistance.

Susceptible host plants are not completely vulnerable because their immune system can combat the vast majority of attackers [4]. Resistance to disease can be described on several levels to stop pathogens from developing in host tissues after infection based on physical and biochemical factors [5]. One type of biochemical response that is strongly associated with defense is the accumulation of phytoalexins, which are defined as low-molecular-weight antimicrobial compounds that are produced after infection [6]. Phytoalexins, which are chemically diverse, include simple phenylpropanoid derivatives, flavonoid- and isoflavonoid-derived phytoalexins, sesquiterpenes, polyketides, etc. [5]. The production of several phytoalexins in *Pinus strobus* after the infection of *B. xylophilus* [7, 8] suggests that the host-pathogen interaction is involved in triggering phytoalexin biosynthesis. However, it is indisputable that *B. xylophilus* can withstand phytoalexins and survive. We believe that some anti-phytoalexin genes regulate nematode resistance to phytoalexins. Genomics research has identified many *B. xylophilus* stress response-related genes [9]. However, whether all of these genes are anti-phytoalexins requires further study. Thus, in this study, we investigated the anti-phytoalexin gene transcript patterns by constructing and sequencing 4 cDNA libraries from *B. xylophilus*. We were particularly interested in determining whether *Bx-cathepsin W* functions in the anti-phytoalexin process of *B. xylophilus* in response to the stress of the pine host of *P. massoniana* and the botanical nematicide carvone. This study presents our investigations into the mechanism of *B. xylophilus* against phytoalexin and the molecular characterization of a *Bx-cathepsin W* gene.

## Methods

### Induction of *B. xylophilus* gene expression by *P. massoniana*

*B. xylophilus* nematodes (collected in GZ, China) were cultured on *Botrytis cinerea* at 25 °C in the dark. A Baermann funnel was used to extract the nematodes (male, female, and juvenile mixed together at a ratio of 1:1:2). To characterize the *B. xylophilus* gene transcript patterns induced by the host *P. massoniana*, a nematode suspension (10,000 nematodes in 500 μl ddH_2_O/seedling) was inoculated into 3-year-old *P. massoniana* seedlings. The suspension was pipetted into an Eppendorf tube, which was filled with degreasing cotton, and inoculated into an artificial wound (1 cm long by 0.5 cm wide) on the seedling. The wound was made by cutting and peeling back the bark to expose the xylem [10]. Three days after inoculation, nematodes were isolated from *P. massoniana* seedlings and then frozen in a mortar with liquid nitrogen and powdered using a pestle for RNA extraction and transcriptome sequencing (set as Treatment 1). An equal number of nematodes cultured on *B. cinerea*, set as a control (CK1), were also powdered.

### Induction of *B. xylophilus* gene expression by Carvone

An assay was performed to characterize the gene transcript patterns of the *B. xylophilus* genes induced by carvone, set as Treatment 2. Carvone, an effective inhibitor of *B. xylophilus* motility, was isolated from the essential oils of *P. sylvestris* in a preliminary trial*.* Stock solutions of carvone were prepared by dilution with 70% ethanol. Working solutions were obtained by diluting the stock solutions with distilled water containing the polysorbate surfactant 20 (Tween-20) [11]. Treatment 2 was performed in the dark at 25 °C in 24-well polystyrene culture plates containing 500 μl carvone (1600 mg/L) per well or ddH_2_O containing ethanol (0.8% v/v) and Tween-20 (0.3% v/v) as CK2. Nematodes (10,000 nematodes in 500 μl ddH_2_O) were transferred to each well and treated for 1 day. Nematodes in Treatment 2 or CK2 were then collected and frozen in a mortar with liquid nitrogen and powdered for RNA extraction and transcriptome sequencing.

### Transcriptome sequencing and differentially expressed gene identification

Total RNA from treated *B. xylophilus* (Treatment 1 and Treatment 2) and CKs (CK1 and CK2) was extracted from the nematode powder using TRIzol (Invitrogen, Carlsbad, CA, USA) and then cleaned using an RNeasy Minikit column (Qiagen, Valencia, CA, USA). The OD_260 nm/_OD_280 nm_ and OD_260 nm_/OD_230 nm_ values of the extracted RNA were quantified using a NanoDrop (Thermo Scientific, Wilmington, DE, U.S.A.). Then, 4 cDNA libraries representing Treatment 1, CK1, Treatment 2, and CK2 were prepared [12] and sequenced by BGI-Shenzhen (Shenzhen, Guangdong, China) according to the BGISEQ-500 standard protocol (Additional file 1: Text S1 and S2) [13, 14]. To analyze the differential expression profiles, 3 biological replicates were performed. The assembly of the clean reads and the quality of the assembly were critically assessed by BGI-Shenzhen (Shenzhen, Guangdong, China) before subsequent analysis (Additional file 1: Text S3). Clean reads were mapped to the reference genome of *B. xylophilus* (BioSample: SAMEA2272519), which is available at NCBI (http://www.ncbi.nlm.nih.gov/assembly/310678), using HISAT2 (v2.0.4, http://www.ccb.jhu.edu/software/hisat) [15]. The dataset was deposited in the Sequence Read Archive (SRA).

To identify the differentially expressed *B. xylophilus* genes, we normalized (by the mean of transcripts per million clean reads, TPM) the read distribution for the gene expression levels in each library to construct an effective library size. The differential gene expression was analyzed according to the method of Li (Additional file 1: Text S4) [16]. The differential gene expression levels were compared using the log_2_(Treatment 1/CK1) or log_2_(Treatment 2/CK2) fold changes of the normalized reads. After the dispersion of each gene was estimated, differentially expressed genes were identified by DESeq using adjusted false discovery rate (FDR) [17]. The threshold of differentially expressed genes was set to log_2_fold change> 1 or < − 1 (FDR < 0.05). The threshold of significant differentially expressed genes was set to log_2_fold change> 1 or < − 1 (FDR < 0.01).

### Identification of anti-phytoalexin genes

The differentially expressed *B. xylophilus* genes induced by the host *P. massoniana* and the botanical nematicide carvone were designated *P. massoniana***-**induced genes and carvone-induced genes, respectively.

The genes that were induced by both *P. massoniana* and carvone and had similarly elevated expression trends were considered anti-phytoalexin genes. Since *B. xylophilus* must face not only phytoalexin but also a more complex mixture of compounds in the resin canals of its pine hosts, carvone-induced *B. xylophilus* genes were used to filter *P. massoniana***-**induced genes to obtain anti-phytoalexin genes. The union of the promoted *P. massoniana***-**induced genes and promoted carvone-induced genes were designated as the anti-phytoalexin genes in this study. Kyoto Encyclopedia of Genes and Genomes (KEGG) analysis and enrichment were performed for the anti-phytoalexin genes.

### *Bx-cathepsin W* cloning and Bx-cathepsin W modeling

Total RNA was extracted from the powder of Treatment 2 nematodes by using TRIzol (Invitrogen, Carlsbad, CA, USA), and the AMV reverse transcription system (Promega, Madison, WI, USA) was subsequently applied to obtain the first-strand cDNA. *Bx-cathepsin W* was then cloned using the first-strand cDNA as the template with the primer pair 5′ GCC TCC GAT CAT GCG ACT AT 3′ and Oligo (dT)_18_. The deduced amino acid sequence of Bx-cathepsin W was submitted to the SWISSModel server [18] for comparative protein structure modeling. Three-dimensional structure alignments of the predicted Bx-cathepsin W were prepared with the program PyMOL [19].

### RNAi of Bx-cathepsin W

Fluorescent siRNAs corresponding to *Bx-cathepsin W* (siRNA sequence: 5′ GGG UGG GCC GUC GAC CAC GGC GUU CUG AC [dT] [dT] 3′, labeled with 5-carboxyfluorescein (FAM) and named *Bx-cathepsin W* RNAi), or *Bx-Actin* (siRNA sequence: 5′ GCC UUC UUU CUU GGG UAU GGA AUC UGC CG [dT] [dT] 3′, labeled with cyanine dyes 3 (Cy3) and named *Bx-Actin* RNAi) were constructed separately. As a nontargeting RNAi treatment, a nontargeting siRNA (siRNA sequence: 5′ AGG AGC UGU UCA CCG GGG UGG UGC CCA UCC U [dT] [dT] 3′) was selected.

RNAi was performed using mixed stages of nematodes soaked in M9 buffer with 10 mM octopamine and siRNAs (2 μg/μl) or M9 buffer with 10 mM octopamine as a control overnight in the dark at 25 °C, as outlined in Wang, et al. [3]. The RNAi-treated and control nematodes were divided into 3 groups. The first group was used to determine the extent of RNAi by real-time quantitative PCR (RT-qPCR) and photographed under a fluorescence microscope (Olympus Bx51, Japan). RT-qPCR was performed with a GoTaq 2-Step RT-qPCR System Kit (Promega, Madison, WI, USA) and a Stratagene Mx3000P qPCR system (Agilent, Santa Clara, CA, USA) to validate the transcript levels of *Bx-cathepsin W* with the primers BxCWqF (TTG CAT TCT ACG GCC AGT CC) and BxCWqR (ACT GAC TTT CGA TGG CTC CG). *Bx-Actin* RNA was used as an internal control with the primers BxACqF (TTC AGG TGT TAC CCA CAC CG) and BxACqR (GCG GTG GTG GTG AAA GAG TA). Each treatment was performed on three separate biological replicates, and each replicate was measured three times. The quantification and normalization of the data followed the log_2_fold method according to the Promega instructions. Student’s *t*-test was used to analyze differences between group grades. The second group was used to assess survival differences. The survival assay of *B. xylophilus* was performed in 24-well polystyrene culture plates containing 500 μl ddH_2_O or carvone (800 mg/L) per well in the dark at 25 °C. One thousand nematodes were then added to each well. Moving and immobilized nematodes were counted every 2 days. The third group was used to assess the pathogenicity difference of *P. massoniana*. RNAi-treated nematodes were inoculated into 3-year-old *P. massoniana* seedlings (10,000 *Bx-cathepsin W* RNAi nematodes in 0.5 ml ddH_2_O/seedling) to test the pathogenicity of *P. massoniana*. The inoculations of M9 buffer-treated nematodes and ddH_2_O were set as the control. Thirty days after inoculation, nematodes were isolated and counted from *P. massoniana* seedlings. Three biological replicates were performed for the inoculation.

### Correlation of *Bx-cathepsin W* to the susceptibility of pines

Weighted gene coexpression network analysis (WGCNA) is a method frequently used to explore the complex relationships between genes and phenotypes [20]. The distinct advantage is that WGCNA transforms gene expression data into coexpression modules, providing insights into signaling networks that may be responsible for phenotypic traits of interest [21]. Therefore, in this study, we aimed to construct coexpression modules using the expression data of different pine-induced *B. xylophilus* genes. The module to which *Bx-cathepsin W* belonged and the association between its expression level and the susceptibility of different species of pines were identified. The survival rate of *B. xylophilus* and the disease susceptibility index of different species of pines (*P. thunbergii* > *P. massoniana > P. sylvestris* var*. mongolica*, our unpublished data) were selected as two traits for WGCNA.

Altogether, 6 transcriptomes (from nematodes induced by *P. massoniana* for 1 day and 15 days, *P. thunbergii* for 1 day and 15 days, *P. sylvestris* var*. mongolica* for 15 days, and CK2, a control of nematodes cultured on *B. cinerea*) were sequenced and analyzed according to the same method described above and were designated as the second sequencing. Two transcriptomes from the first sequencing (Treatment 1 and CK1) and all 6 transcriptomes from the second sequencing were analyzed by weighted gene WGCNA to find clusters (modules) of highly correlated anti-phytoalexin genes (Additional file 1: Text S5) [22]. The appropriate power value was determined when the degree of independence was over 0.8. The minimum number of genes was set as 30 for the high reliability of the results. For each expression profile, gene significance (GS) was calculated as the absolute value of the correlation between expression profile and each trait; module membership (MM) was defined as the correlation of expression profile and each module eigengene.

## Results

### Transcriptome sequencing and differentially expressed genes identification

To characterize the anti-phytoalexin gene transcript patterns, 4 cDNA libraries (Treatment 1, CK1, Treatment 2, and CK2) of *B. xylophilus* were constructed and sequenced. The dataset was deposited in the SRA (Accession No. SRS3880513, NCBI Submission ID: SUB4555785, and BioProject ID: PRJNA494797).

An average of 79.69 Mb raw reads was obtained for each sample. After removing the low-quality reads, more than 66.74 Mb clean reads (6.62 Gb clean bases) for each sample were obtained. Clean reads were mapped to the reference genome, the result showed that more than 93% of the transcripts could mapping to the reference genome (Additional file 1: Table S1) [9]. A total of 17,747 assembled unigenes were generated from the 4 libraries. On the basis of differential expression analysis [23] and the FDR [17], 2051 differentially expressed genes (including 784 significant differentially expressed genes) and 15,696 unchanged or nonsignificant differentially expressed genes were found from the Treatment 1 and CK1 libraries. Meanwhile, 1452 differentially expressed genes (including 715 significant differentially expressed genes) and 16,295 unchanged or nonsignificant differentially expressed genes were found from the Treatment 2 and CK2 libraries.

### Anti-phytoalexin genes identification

**Fig. 1:**
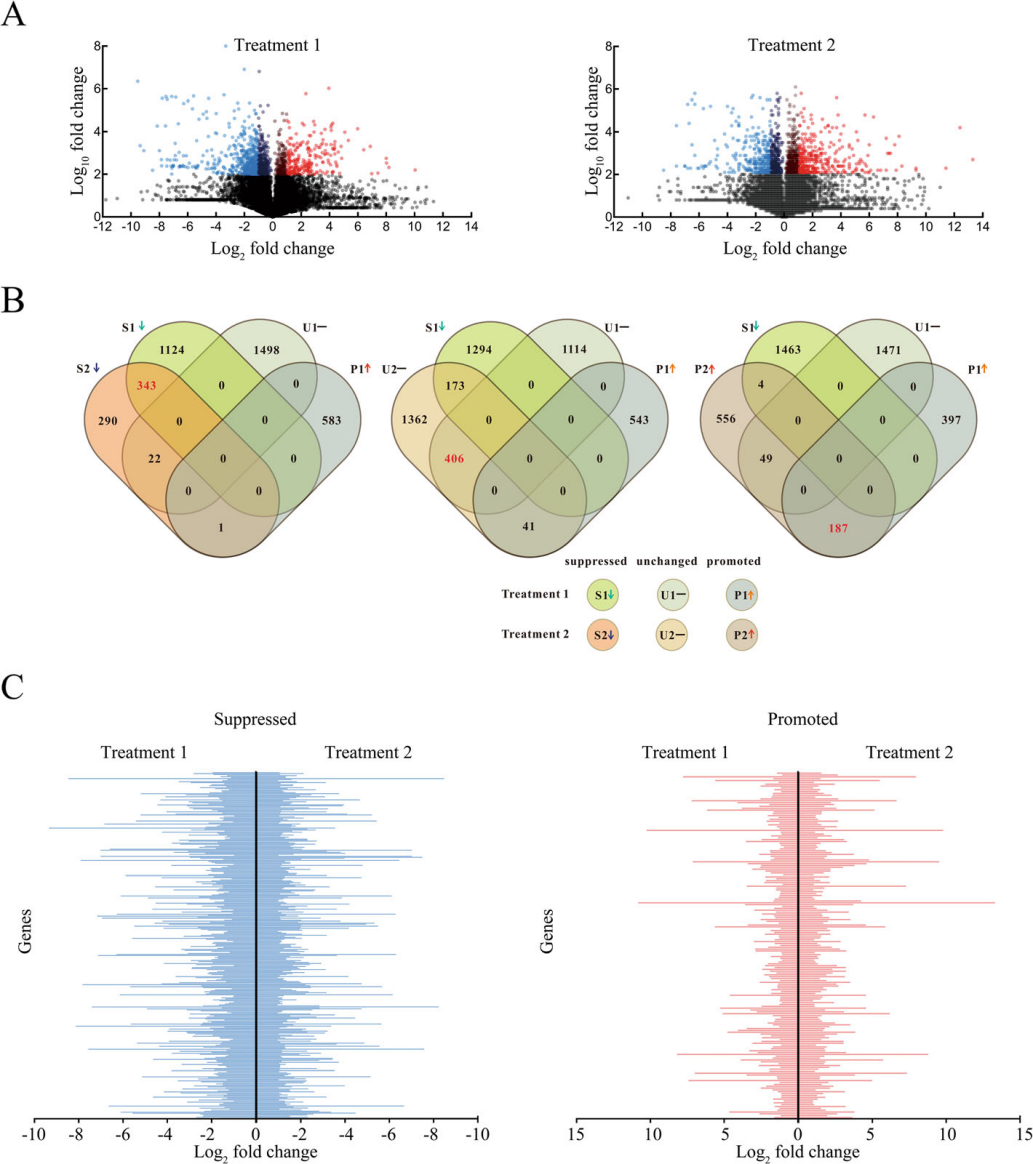
Identification of carvone- and *P. massoniana*-induced genes (all figures were generated by the authors of this study). **a**. Scatter plot of the gene expression of *P. massoniana*-induced and carvone*-*induced genes in Treatment 1 and Treatment 2 libraries according to the log_2_fold change of normalized reads and the -log_10_fold change of the *p*-value (*t*-test, *n* = 3). Expression levels are normalized by the mean numbers of transcripts per million clean reads (TPM). Data points colored blue, red or black represent suppressed genes, promoted genes or nonsignificant differentially expressed genes. **b**. The intersections of lists of *P. massoniana*-induced and carvone*-*induced genes. The Venn diagram displays the distribution of 3571 unique genes from Treatment 1 and 3434 unique genes from Treatment 2. S1 and S2 represent suppressed genes (log_2_fold < − 1, *p* < 0.05 and FDR < 0.05). U1 and U2 represent unchanged genes (− 1 < log_2_fold < 1 or *p* > 0.05). P1 and P2 represent promoted genes (log_2_fold > 1, *p* < 0.05 and FDR < 0.05). **c**. Profiles of genes that show the same expression change trends when induced by both *Pinus massoniana* and carvone. Treatment 1: Induction of *B. xylophilus* gene expression by *P. massoniana*. Treatment 2: Induction of *B. xylophilus* gene expression by carvone

A total of 2051 *P. massoniana***-**induced genes were found, including 584 promoted and 1467 suppressed genes (Fig. 1a Treatment 1). Furthermore, 1452 carvone-induced genes were identified, including 796 promoted and 656 suppressed genes (Fig. 1a Treatment 2). The results indicated that more genes responded to induction by *P. massoniana* than induction by carvone, which might be because *B. xylophilus* has to face a more complex mixture of compounds in the resin canals of pine hosts. Therefore, we filtered anti-phytoalexin genes by intersections of lists of *P. massoniana*-induced genes and carvone-induced genes. Altogether, 187 anti-phytoalexin genes were identified. Additionally, we identified 343 suppressed genes that showed the same expression patterns when induced by both *Pinus massoniana* and carvone (Fig. 1b), and the expression of those promoted and suppressed genes is detailed in Fig. 1c.

Further analysis showed that the 187 anti-phytoalexin genes could be divided into 4 groups. The 1st group included 29 pioneer genes, which had no homolog in either the NCBI Nonredundant sequences (nr) database or the genome of *Caenorhabditis elegans*. The 2nd group included 84 genes that had a homolog in the nr database (including 23 hypothetical proteins or uncharacterized proteins) but had no homolog in the genome of *C. elegans*. The 3rd group included 54 genes that had a homolog in the genome of *C. elegans*. The 4th group included 20 xenobiotic-metabolizing enzyme (XME) genes (Additional file 1: Table S2).

Among those 187 anti-phytoalexin genes, 95 genes could be enriched as 66 KEGG orthologs (Additional file 1: Table S3), 33 genes were enriched as Lysosome pathway especially (Additional file 1: Table S4), 4 anti-phytoalexin genes were enriched as cathepsins (K01373 or K01365), which were in the pathways Lysosome (ko04142), Phagosome (ko04145), Antigen processing and presentation (ko04612), and Apoptosis (ko04210). Since cathepsins help to maintain metabolic homeostasis by participating in the degradation of heterophagic and autophagic material [24], homologous Cathepsin W-encoding genes were studied.

### Identification of homologous Cathepsin W-encoding genes

**Fig. 2:**
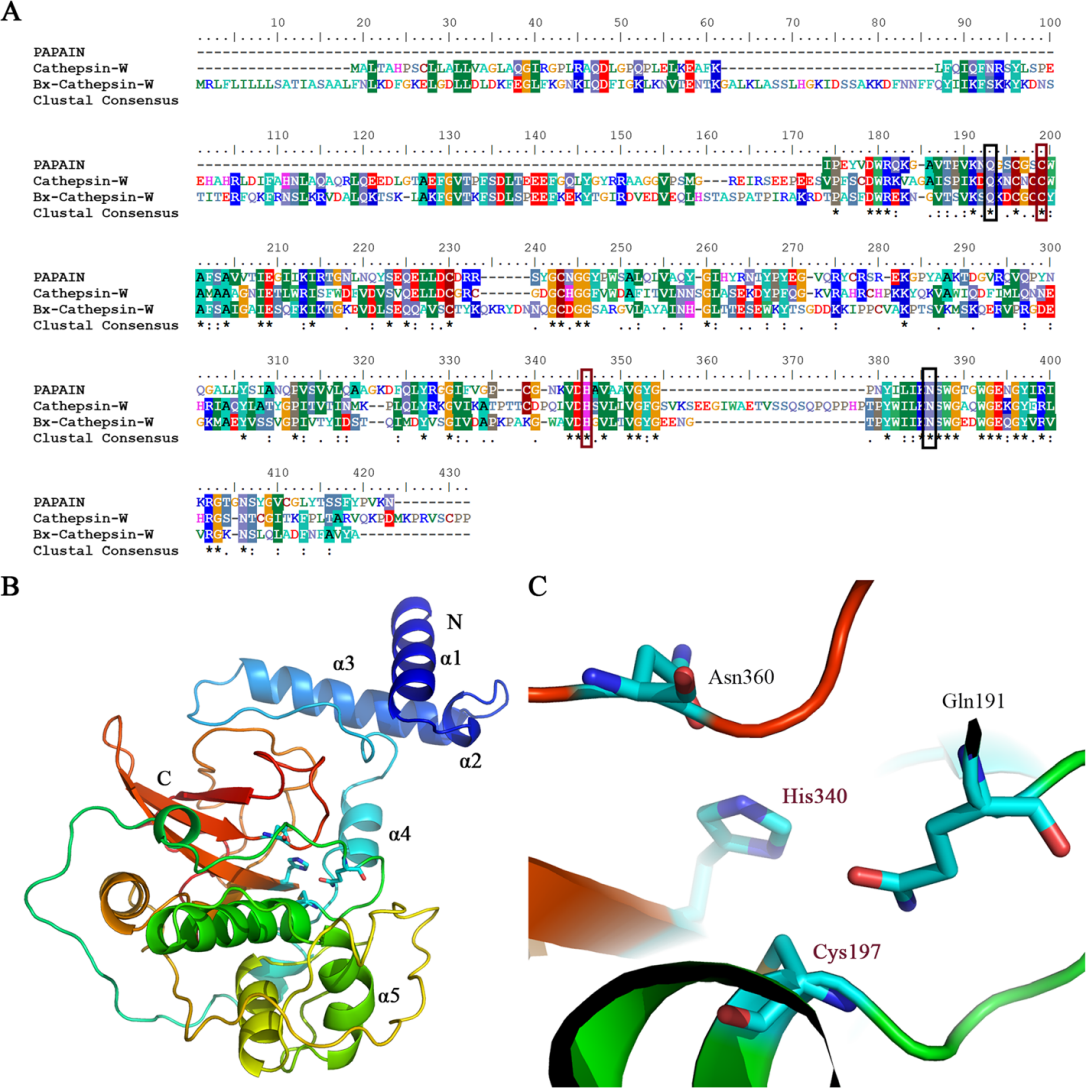
Alignment and structure of Bx-cathepsin W (all figures were generated by the authors of this study). **a**. Multiple-sequence alignment of Bx-cathepsin and homologs (papain and cathepsin W). Highly conserved residues of the catalytic triad are labeled as red boxes. The residues that play an important role in catalysis are labeled as black boxes. **b**. The dimeric structure of Bx-cathepsin W. The N-terminal α-helices are labeled α1~α5. **c**. The dimeric structure of the catalytic active site

A cDNA for a homolog of a cysteine protease, designated *Bx-cathepsin W* (BUX.s00600.106), was cloned from the Treatment 2 cDNA library. *Bx-cathepsin W* contained a coding sequence of 1179 bp, encoding a polypeptide of 392 amino acids composed of a putative 16-residue signal peptide, 141-residue propeptide, and a 235-residue mature protein. Protein sequence comparisons revealed 77% homology with human cathepsin W (E value = 3e-43) [25], 76% homology with *C. elegans* cathepsin F (E value = 3e-49), and approximately 53~75% homology with human cathepsins F, O, X, K, H, and S (E value = 3e-45~1e-39). The deduced amino acid sequence contained the highly conserved residues of the catalytic triad of papain-like cysteine proteases (Fig. 2a). The results indicated that the protein belonged to the peptidase C1A subfamily (MEROPS database nomenclature), which was composed of cysteine peptidases that were similar to papain. The highly conserved residues of the catalytic triad (Cys197 and His340) formed a catalytic dyad (Fig. 2a and c). Two other residues played important roles in catalysis: a Gln191 preceding the catalytic Cys197 and an Asn360 residue that orients the imidazolium ring of the catalytic His340 (Fig. 2a and c). The crystal structure of Bx-cathepsin W was typical of C1A cysteine proteases. The N-terminal propeptide contained 5 α-helices (Fig. 2b α1~α5) and was in close contact with the mature domain, which comprised 5 α-helices and a β-sheet formed from 4 antiparallel β-strands.

### RNAi of Bx-cathepsin W

**Fig. 3:**
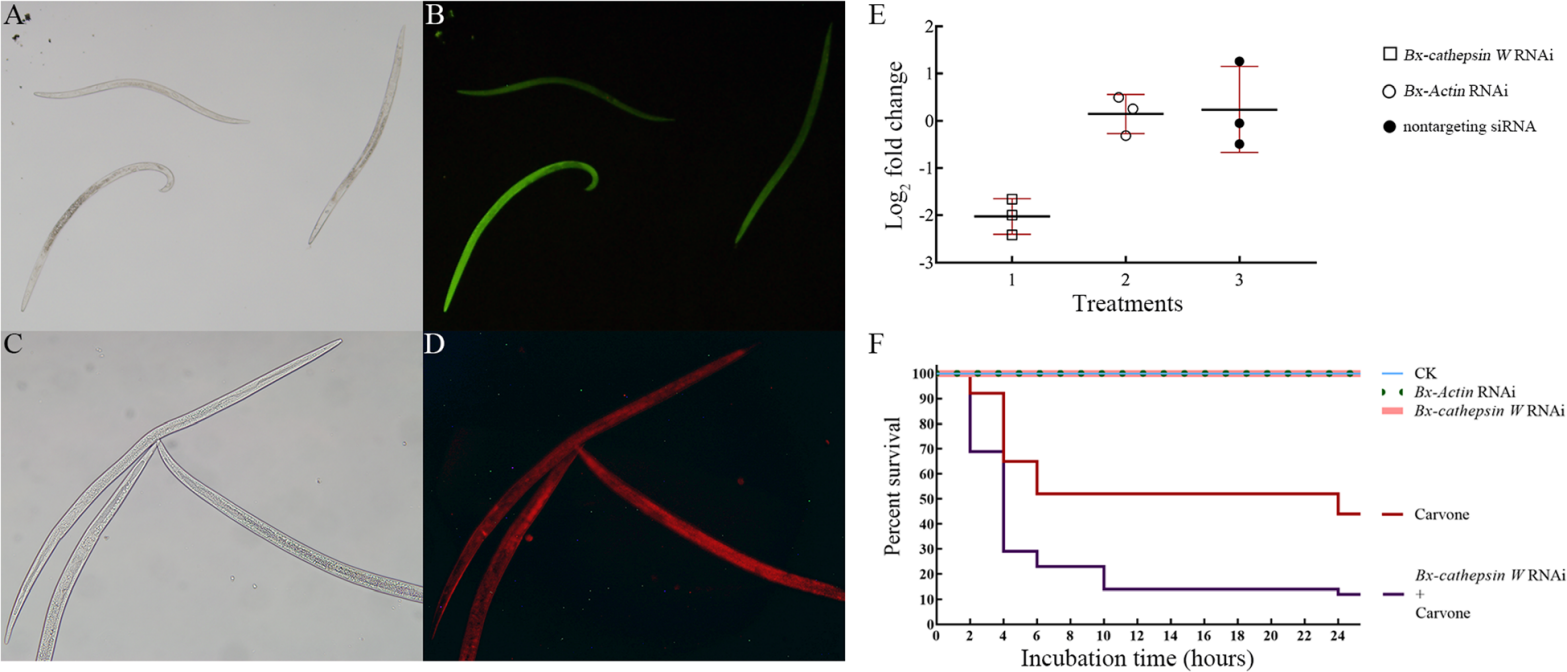
*Bx-cathepsin W* RNAi in *B. xylophilus* (all figures were generated by the authors of this study). **a**. Morphology of *Bx-cathepsin W* RNAi-treated *B. xylophilus*. **b**. The green fluorescence indicates the FAM-labeled *Bx-cathepsin W* corresponding with effective siRNA entry into the nematode. **c**. Morphology of *Bx-Actin* RNAi-treated *B. xylophilus*. **d**. The red fluorescence indicates the Cy3-labeled *Bx-Actin* corresponding with effective siRNA entry into the nematode. **e**. The expression of *Bx-cathepsin W* detected by RT-qPCR after soaking in the relevant siRNA of *Bx-cathepsin W*, *Bx-Actin*, and nontargeting. **f**. Percent survival of *Bx-cathepsin W* RNAi- and carvone-treated *B. xylophilus*

A potent and specific *Bx-cathepsin W* silencing was found in *B. xylophilus* after the nematode soaked in siRNAs overnight. The patterns of fluorescein observed for the nematodes indicated that FAM/Cy3 labeled siRNA was taken up by *B. xylophilus* effectively (Fig. 3b and d). RT-qPCR showed significant suppression of *Bx-cathepsin W*, whereas there was no mutual interference between the two genes of *Bx-cathepsin W* and *Bx-Actin* after soaking in the relevant siRNA for 12 h (Fig. 3e). For the expression of *Bx-cathepsin W*, no significant difference between the *Bx-Actin* siRNA and nontargeting siRNA buffer was found after soaking for 12 h. None of the RNAi-treated nematodes presented a significant difference in survival rate (Student’s *t*-test, *n* = 5, bivariate correlation analysis, *Bx-cathepsin W* RNAi *p* = 0.68, *Bx-Actin* RNAi *p* = 1.00) and morphology (Fig. 3a and c) compared to the M9 buffer-treated group during the whole 10-day study.

However, the survival rate of the *Bx-cathepsin W* RNAi-treated nematode was reduced significantly after being soaked in the carvone solution compared to that of the M9 buffer-treated nematodes (Fig. 3f. Student’s *t*-test, n = 5, bivariate correlation analysis, *p* = 0.00). This result indicated that the resistance of *B. xylophilus* to carvone decreased after the knockdown of *Bx-cathepsin W*.

**Fig. 4:**
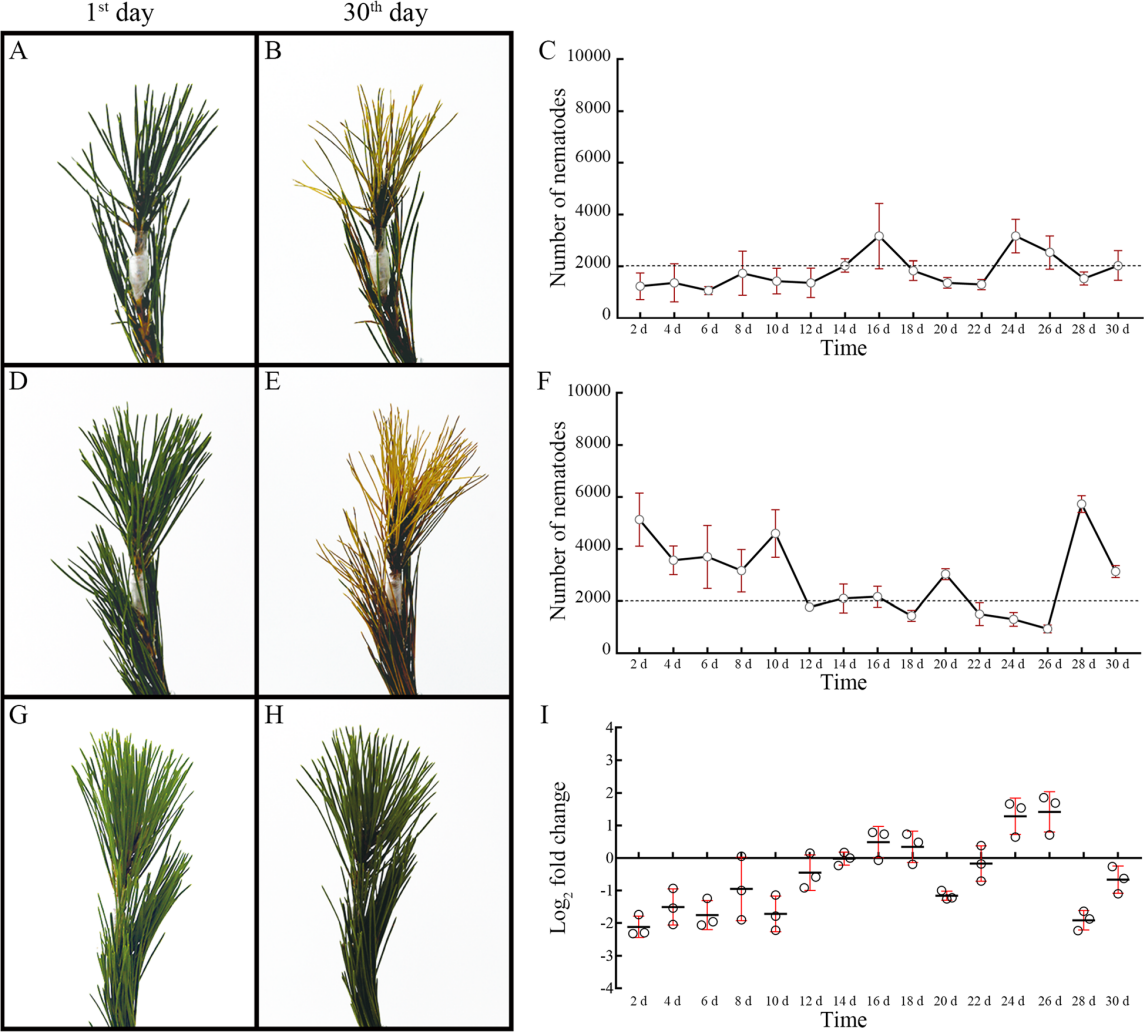
Symptoms of *P. massoniana* at the 30th day post-inoculation with wild type and *Bx-cathepsin W* RNAi-treated *B. xylophilus* (all figures were generated by the authors of this study). **a**. Symptoms at the 1st day post-inoculation with *Bx-cathepsin W* RNAi-treated *B. xylophilus*. **b**. Symptoms at the 30th day post-inoculation with *Bx-cathepsin W* RNAi-treated *B. xylophilus*. **c**. The number of nematodes isolated from *P. massoniana* inoculated with the *Bx-cathepsin* RNAi-treated nematodes. **d**. Symptoms at the 1st day post-inoculation with M9 buffer-treated *B. xylophilus*. **e**. Symptoms at the 30th day post-inoculation with M9 buffer-treated *B. xylophilus*. **f**. The number of nematodes isolated from *P. massoniana* inoculated with the M9 buffer-treated nematodes. **g**. Symptoms at the 1st day post-inoculation of ddH_2_O. H. Symptoms at the 30th day post-inoculation with ddH_2_O. **i**. The log_2_fold changes of the number of nematodes isolated from *P. massoniana* inoculated with the *Bx-cathepsin W* RNAi-treated nematodes and from *P. massoniana* inoculated with M9 buffer-treated nematodes)

More importantly, significant pathogenicity attenuation in the *Bx-cathepsin* RNAi-treated nematodes was found (Fig. 4). Significant variations occurred in seedlings inoculated with *Bx-cathepsin W* RNAi-treated *B. xylophilus* (Fig. 4f) compared with the M9 buffer-treated nematodes (Fig. 4e). After 30 days, seedlings inoculated with the *Bx-cathepsin W* RNAi-treated *B. xylophilus* were not completely wilted; in contrast, all seedlings inoculated with M9 buffer-treated nematodes were completely wilted, while no wilt symptoms were found in the ddH_2_O-inoculated seedlings. After 30 days, few nematodes could be isolated from the *Bx-cathepsin* RNAi-treated nematode inoculation (Fig. 4c, f, and i). This result indicated that the knockdown of *Bx-cathepsin W* reduced the survival rates of *B. xylophilus* under *P. massoniana* stress.

### Correlation of *Bx-cathepsin W* to the susceptibility of pines

**Fig. 5:**
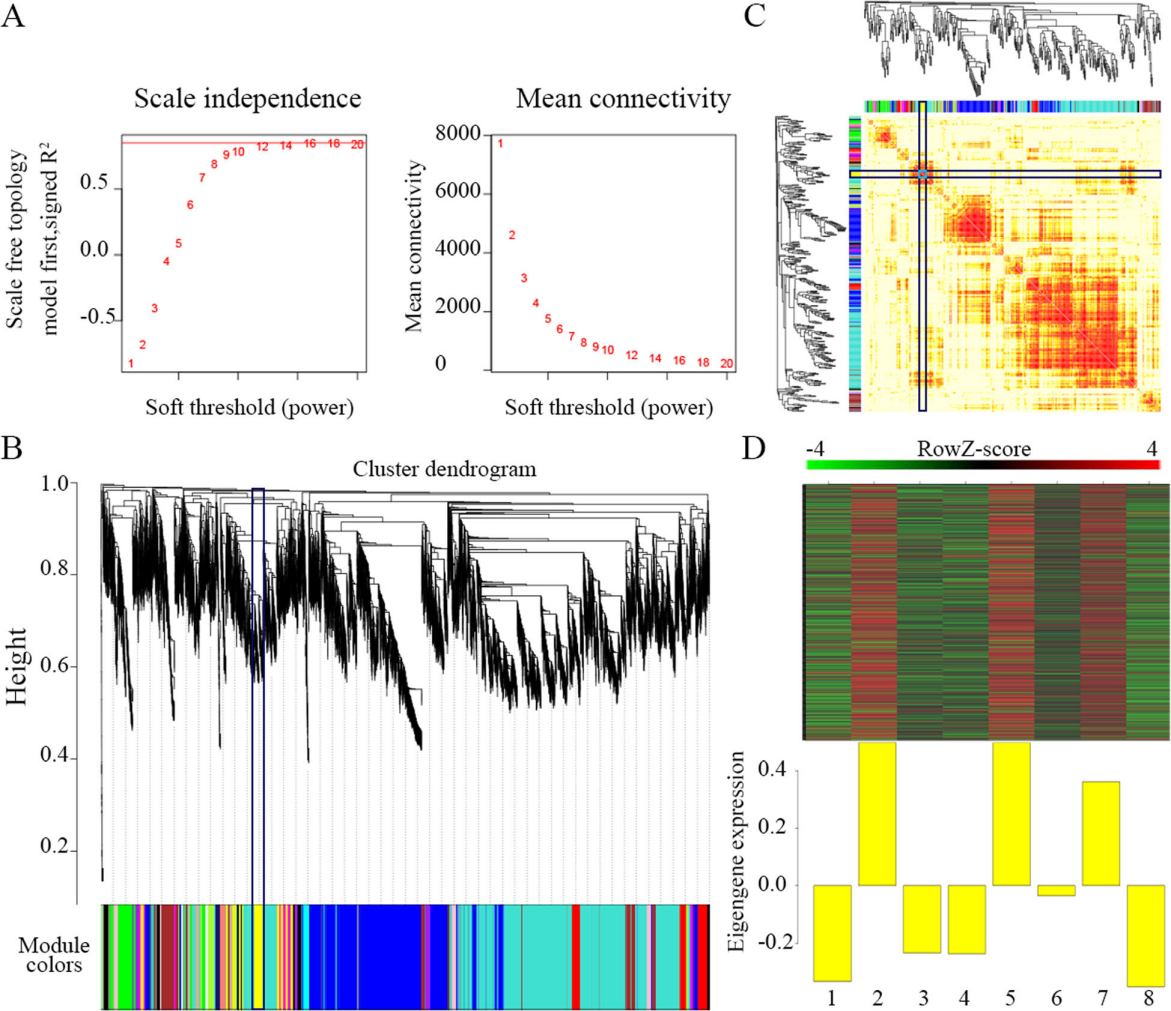
WGCNA analysis of 8 *B. xylophilus* transcriptomes (all figures were generated by the authors of this study). **a**. Analysis of network topology for various soft-thresholding powers. The left panel shows the scale-free fit index (y-axis) as a function of the soft-thresholding power (x-axis). The right panel displays the mean connectivity (degree, y-axis) as a function of the soft-thresholding power (x-axis). **b**. Clustering dendrograms of genes with dissimilarity based on the topological overlap, together with assigned module colors. As a result, 25 coexpression modules were constructed and are shown in different colors. **c**. Visualizing the gene network using a heatmap plot. The heatmap depicts the topological overlap matrix among all genes in the analysis. Light color represents low overlap, and the progressively darker red color indicates increased overlap. Blocks of darker colors along the diagonal are the modules. The gene dendrogram and module assignment are also shown along the left side and the top. **d**. 1: CK2, 2: nematodes induced by *P. massoniana* for 1 day, 3: nematodes induced by *P. massoniana* for 15 days, 4: nematodes induced by *P. sylvestris* var*. mongolica* for 15 days, 5: nematodes induced by *P. thunbergii* for 1 day, 6: nematodes induced by *P. thunbergii* for 15 days, 7: Treatment 1, 8: CK1

**Fig. 6:**
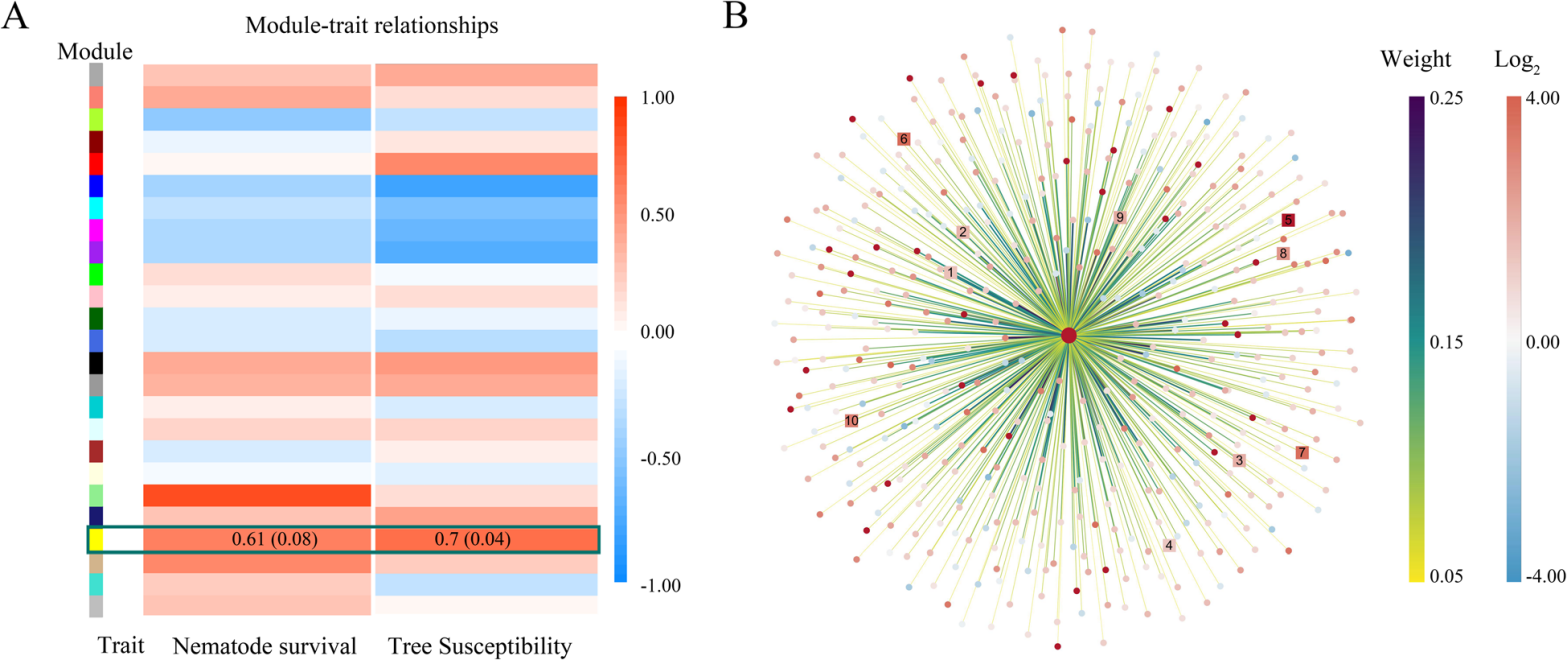
Correlation of *Bx-cathepsin W* to the susceptibility of pines and other genes in the yellow module (all figures were generated by the authors of this study). **a**. Module-trait associations. Each row corresponds to a module eigengene, and each column corresponds to a trait. The table is color-coded by correlation according to the color legend. **b**. Interaction of 439 *Bx-cathepsin W*-correlated genes identified from the yellow module. Each dot and square corresponds to a gene, with *Bx-cathepsin W* at the center and the squares representing 10 anti-phytoalexin genes. The colors of the dots and squares represent the range of the gene’s expression, log_2_ represent log_2_(Treatment 1/CK1)). The thickness and color of the lines represent connections of corresponding topological overlap

Based on the initial results, expression values of 17,624 genes in 8 transcriptomes of *B. xylophilus* were used to construct the coexpression module with WGCNA package tools. First, the power value was screened out (Fig. 5a). When the power value was equal to 12, the independence degree was up to 0.82, and the average connectivity degree was higher. Therefore, this power value was used to construct coexpression modules, and 25 distinct gene coexpression modules were identified in *B. xylophilus*. These coexpression modules were constructed and shown in different colors, and the number of genes in the 25 modules is shown in Additional file 1: Table S5 (Fig. 5b). Interactions of the 25 coexpression modules were analyzed (Fig. 5c). Modules with common expression pattern interaction analysis of coexpression modules that were associated with particular traits were identified based on the correlation between module eigengene and traits (Fig. 6a). Genes in the yellow module show different expression patterns for different time points. The expression of most genes increased when *B. xylophilus* was inoculated into pine trees for 1 day (Fig. 5d bar 2 and 5) or 3 days (Fig. 5d bar 7), and few genes were promoted at 15 days (Fig. 5d bar 3, 4 and 6).

The survival rate of *B. xylophilus* and the disease susceptibility index of different species of pine were selected for the two traits. Interaction analysis of coexpression modules that were associated with particular traits were identified based on the correlation between module eigengene and trait (Fig. 6a). The module eigengene is defined as the first principal component of a given module. It can be considered a representative of the gene expression profiles in a module. The expression profile of the eigengene of the yellow module, to which *Bx-cathepsin W* belongs, was positively correlated to the survival rate of the nematodes (correlation: 0.61, and *p*-value: 0.08) and pine susceptibility (correlation: 0.7, and *p*-value: 0.04), which indicated that genes in this module were related to the interaction between nematodes and pines. The GS of *Bx-cathepsin W* with survival rate was 0.79 (*p*-value: 0.01), the GS of *Bx-cathepsin W* with pine susceptibility was 0.77 (*p*-value: 0.01), and the MM of *Bx-cathepsin W* with yellow module was 0.79 (*p*-value: 0.01). This result indicated that *Bx-cathepsin W* had a strong positive correlation with the susceptibility of pines; in other words, *Bx-cathepsin W* could improve the anti-phytotoxin ability of *B. xylophilus*, which was consistent with the *Bx-cathepsin W* RNAi analysis and suggested that *Bx-cathepsin W* functions as an anti-phytotoxin via an additional method of analysis.

Additionally, 661 *Bx-cathepsin W*-correlated genes were identified from the yellow module; among them, the connections of the corresponding topological overlap of 439 genes were above a threshold of 0.08 (Fig. 6b). Further analysis of these 439 genes indicated that there were 10 *Bx-cathepsin W*-correlated anti-phytoalexin genes (Additional file 1: Table S6). The *Bx-cathepsin W*-correlated anti-phytoalexin genes also had 4421 interactions with additional 812 genes.

## Discussion

The stress response is an essential process for *B. xylophilus* survival. Genomics research has identified many *B. xylophilus* stress response-related genes, such as the glutathione S-transferase [26], uridine diphosphate glucuronosyl transferase, cytochrome P450, and abscisic acid transporter gene families [9]. In this study, we compared *B. xylophilus* transcriptomes with the *B. xylophilus* genome and *C. elegans* genome to identify stress response-related genes, whereas the result indicated that not all of these genes were stimulated (Additional file 1: Table S7) [9, 27, 28]. For example, only 68 cytochrome P450 monooxygenase genes and 35 xenobiotic transport across cell membranes related ATP-binding cassette transporter were found. This is consistent with previous transcriptomic studies about *B. xylophilus* parasitic adaptation to life on pine hosts, also only 48 cytochrome P450 family members were found [29]. Beyond this, 5 more UDP glucuronosyltransferases transcript were found, suggesting that there may be multiple splicing patterns. Previous transcriptome studies have also shown that only 12 genes were involved in the xenobiotic metabolism while the nematode transition from mycetophagous (fungal feeding) to phytophagous phase (nematodes infected to host) [30].

Furthermore, in order to find a common core among different phytoalexins induced *B. xylophilus* defense genes, all 187 anti-phytoalexin genes were compared with other *B. xylophilus* transcriptome studies [31, 32]. However, only 2 genes (one small GTPase gene BUX.s01147.18 and one peroxidase gene BUX.c09083.1) were found to overlap with 41 DEGs induced by α-pinene. And, only 1 gene (one peptidase gene BUX.s00422.693) were found to overlap with 30 DEGs between mycophagous and phytophagous stages *B. xylophilus* transcriptome. The DEGs between mycophagous and phytophagous reveal that *B. xylophilus* need a multilayer detoxification strategy in order to protect itself from host defense responses [32]. Those results indicate that maybe different phytoalexins induce nematode gene expression in its own way. Given the results, stress response-related genes may have more complex functions to allow *B. xylophilus* to cope with complex environments, such as interacting with other hosts, nematode-trapping fungus and other fungi (such as the blue-strain fungus) in the pine or the vector *Monochamus alternatus*. In particular, *B. xylophilus* must cope with phytoalexins, which are induced by pine in response to nematode infection. Anti-phytoalexin is a crucial process for *B. xylophilus* to cope with its pine hosts.

In this study, we identified 187 anti-phytoalexin genes that showed the same promoted expression trends upon induction by both *P. massoniana* and carvone. However, the detailed functions of these anti-phytoalexin genes require further study. The roles of nematode cathepsins, one proteinase of cysteine proteinases (EC 3.4.22), may include invasion of host tissues, parasite nutrition and evasion of host defense responses [33, 34]. Cathepsins are secreted as inactive proenzymes and have an N-terminal propeptide that is cleaved during maturation. Cathepsins L have been identified in the plant parasites *Heterodera glycines* [35] and *Meloidogyne incognita* [33]. Cysteine proteases are responsible for host hemoglobin degradation, as shown in *Necator americanus* [36] and *Teladorsagia circumcincta* [37]. Previous studies have suggested that cathepsins are involved in the digestive processes of adult *H. glycines* [35] and *M. incognita* [33] females. In addition, the function of the cysteine protease in *M. incognita* preparasitic infective J2 s is directly related to the parasitic aspects of the plant-nematode interaction [33].

Therefore, we were particularly interested in determining whether *Bx-cathepsin W* functioned in the anti-phytoalexin process of *B. xylophilus* in response to *P. massoniana* and carvone stresses. Carvone-treated *B. xylophilus* was not strictly correlated with feeding in Treatment 2, and the promotion of *Bx-cathepsin W* expression indicates that some function of *Bx-cathepsin W* is indeed more directly related to the plant-nematode interaction, especially to the anti-phytoalexin process. The results of the RNAi assessment indicated that *Bx-cathepsin W* supported the survival of *B. xylophilus* under carvone or *P. massoniana* stress. From the relationship between the expression level of *Bx-cathepsin W* and the survival rate of *B. xylophilus* in different pines, we found that the gene expression was positively related to the survival rate of *B. xylophilus*. Therefore, the correlation of the expression level of *Bx-cathepsin W* with the susceptibility of pines was examined. These results showed that *Bx-cathepsin W* played an important role in improving the anti-phytotoxin ability of *B. xylophilus*. The survival rate of *B. xylophilus* was positively correlated with the susceptibility of pines; that is, the nematodes that infect more susceptible pines are more likely to survive and therefore are greater in number than nematodes that infect less susceptible pines. Therefore, based on the above results, *Bx-cathepsin W* is important in the pine wood nematode infection process. According to the correlation between genes in the yellow module, genes that shared the same expression pattern and had a high correlation with *Bx-cathepsin W* might have a strong regulatory relationship with *Bx-cathepsin W*, which could indicate a direction for further research on *Bx-cathepsin W* and a new target for the control of the nematode.

## Conclusions

The results indicated that the anti-phytoalexin genes, particularly *Bx-cathepsin W*, support the survival of the nematode *B. xylophilus* under *P. massoniana* phytoalexin stress. The cDNA library sequencing, differentially expressed gene identification and WGCNA algorithm provided insight at a systemic level into the gene regulation of *B. xylophilus* in response to the immune reaction of *P. massoniana*. These results will lead to a better understanding of the function of nematode defenses in host innate immunity.

## Supplementary information

**Additional file 1: Table S1.** Statistical analysis of the RNA sequencing data. **Table S2.** Xenobiotic-metabolizing enzyme genes. **Table S3.** Results of KEGG enrichment. **Table S4.** Anti-phytoalexin genes related to the Lysosome pathway. **Table S5.** The number of genes in the 25 modules. **Table S6.** *Bx-cathepsin W*-correlated anti-phytoalexin genes. **Table S7.** Identification of *B. xylophilus* xenobiotic metabolism genes. **Text S1.** Transcriptome library preparation and sequencing. **Text S2.** Data analysis of reads. **Text S3.** Transcriptome assembly. **Text S4.** The differential gene expression analysis. **Text S5.** The WGCNA algorithm.

## Data Availability

All supporting data are included as additional files as “Supplemental data files.docx”.

## Abbreviations

Cy3: cyanine dyes 3
FAM: 5-carboxyfluorescein
FDR: false discovery rate
GS: gene significance
KEGG: Kyoto Encyclopedia of Genes and Genomes
MM: module membership
Nr: NCBI Nonredundant sequences database
RT-qPCR: real-time quantitative PCR
SRA: Sequence Read Archive
TPM: transcripts per million clean reads
Tween-20: polysorbate surfactant 20
WGCNA: weighted gene coexpression network analysis
XMEs: xenobiotic-metabolizing enzymes
