# Supplementary material for: The anti-phytoalexin gene Bx-cathepsin W supports the survival of Bursaphelenchus xylophilus under Pinus massoniana phytoalexin stress
Source: BMC Genomics. 2019 Oct 26;20:779. doi: 10.1186/s12864-019-6167-2 (PMC6815438; doi:10.1186/s12864-019-6167-2)
Supplement: Supplementary file 1 — Additional file 1: Table S1. Statistical analysis of the RNA sequencing data. Table S2. Xenobiotic-metabolizing enzyme genes. Table S3. Results of KEGG enrichment. Table S4. Anti-phytoalexin genes related to the Lysosome pathway. Table S5. The number of genes in the 25 modules. Table S6. Bx-cathepsin W-correlated anti-phytoalexin genes. Table S7. Identification of B. xylophilus xenobiotic metabolism genes. Text S1. Transcriptome library preparation and sequencing. Text S2. Data analysis of reads. Text S3. Transcriptome assembly. Text S4. The differential gene expression analysis. Text S5. The WGCNA algorithm. [file 12864_2019_6167_MOESM1_ESM.docx]

**The anti-phytoalexin gene *Bx-cathepsin W* supports the survival of *Bursaphelenchus xylophilus* under** ***Pinus massoniana* phytoalexin stress**

Feng Wang^§^, Qiaoli Chen^§^, Ruizhi Zhang, Danlei Li, Yaming Ling, Ruiqing Song^*^

Key Laboratory of Sustainable Forest Ecosystem Management-Ministry of Education, School of Forestry, Northeast Forestry University, Harbin 150040, Heilingjiang, P. R. China

**^§^These authors contributed equally to this work**

^*^**Corresponding authors:**

**Table S1** **Statistical analysis of the RNA sequencing data**

| Samples | Clean Reads  Q20(%) | Clean Reads  Ratio(%) | Gene Mapping  Rate (%) |
| --- | --- | --- | --- |
| Treatment 1-1 | 94.85 | 83.03 | 92.91 |
| Treatment 1-2 | 94.86 | 82.93 | 93.34 |
| Treatment 1-3 | 94.73 | 83.05 | 92.23 |
| Treatment 2-1 | 97.85 | 83.56 | 93.32 |
| Treatment 2-2 | 95.66 | 84.91 | 93.43 |
| Treatment 2-3 | 95.23 | 83.78 | 93.88 |
| CK1-1 | 96.45 | 83.21 | 93.22 |
| CK1-2 | 94.01 | 83.96 | 92.49 |
| CK1-3 | 95.66 | 83.45 | 93.56 |
| CK2-1 | 96.64 | 84.27 | 93.69 |
| CK2-2 | 96.89 | 84.65 | 92.21 |
| CK2-3 | 95.65 | 84.19 | 92.66 |

**Table S2 Xenobiotic-metabolizing enzyme genes**

| Bx gene | Phase | **log_2_-Carvone** | **log_2_-inoculation** |
| --- | --- | --- | --- |
| *Bx-gst29* | Phase II | **1.0** | **1.3** |
| *Bx-dhs21* | Phase I | **1.4** | **1.8** |
| *Bx-gst43* | Phase II | **1.5** | **1.2** |
| *Bx-akr14* | Phase I | **1.5** | **1.7** |
| *Bx-gst7* | Phase II | **1.6** | **2.4** |
| *Bx-ugt5* | Phase II | **1.6** | **1.6** |
| *Bx-alh4* | Phase I | **1.6** | **1.2** |
| *Bx-ugt63* | Phase II | **1.6** | **1.2** |
| *Bx-ugt61* | Phase II | **1.7** | **1.7** |
| *Bx-ugt2* | Phase II | **1.8** | **1.6** |
| *Bx-dhs73* | Phase I | **1.8** | **3.3** |
| *Bx-gst42* | Phase II | **2.3** | **2.4** |
| *Bx-ugt21* | Phase II | **2.5** | **2.1** |
| *Bx-ugt20* | Phase II | **2.6** | **2.2** |
| *Bx-dhs4* | Phase I | **2.6** | **2.6** |
| *Bx-gst33* | Phase II | **3.2** | **2.9** |
| *Bx-ce16* | Phase I | **3.5** | **1.7** |
| *Bx-gst8* | Phase II | **4.6** | **3.4** |
| *Bx-gst2* | Phase II | **5.9** | **5.6** |
| *Bx-gst53* | Phase II | **7.3** | **3.5** |

**Table S3 Results of KEGG enrichment**

| **No** | **KEGG** | **Enriched genes** | **Lysosome related** | **Name** |
| --- | --- | --- | --- | --- |
| 1 | K00011 | 1 | 0 | aldehyde reductase [EC:1.1.1.21] |
| 2 | K00026 | 1 | 0 | malate dehydrogenase [EC:1.1.1.37] |
| 3 | K00128 | 1 | 0 | aldehyde dehydrogenase (NAD+) [EC:1.2.1.3] |
| 4 | K00602 | 3 | 0 | phosphoribosylaminoimidazolecarboxamide formyltransferase / IMP cyclohydrolase [EC:2.1.2.3 3.5.4.10] |
| 5 | K00699 | 5 | 0 | glucuronosyltransferase [EC:2.4.1.17] |
| 6 | K00799 | 8 | 0 | glutathione S-transferase [EC:2.5.1.18];K01830 |
| 7 | K01044 | 1 | 0 | carboxylesterase 1 [EC:3.1.1.1];K01050 |
| 8 | K01052 | 2 | LIPA | lysosomal acid lipase/cholesteryl ester hydrolase [EC:3.1.1.13] |
| 9 | K01373 | 4 | Lysosome | cathepsin F [EC:3.4.22.41];K01365 |
| 10 | K01415 | 1 | 0 | endothelin-converting enzyme [EC:3.4.24.71];K08635 |
| 11 | K01757 | 1 | 0 | strictosidine synthase [EC:4.3.3.2] |
| 12 | K01904 | 1 | 0 | 4-coumarate--CoA ligase [EC:6.2.1.12];K00666 |
| 13 | K01915 | 1 | 0 | glutamine synthetase [EC:6.3.1.2] |
| 14 | K02599 | 1 | 0 | Notch |
| 15 | K03132 | 2 | 0 | transcription initiation factor TFIID subunit 7;K03443 |
| 16 | K03450 | 2 | 0 | solute carrier family 7 (L-type amino acid transporter), other;K13780 |
| 17 | K04650 | 1 | 0 | nuclear receptor co-repressor 1 |
| 18 | K04828 | 1 | 0 | acid-sensing ion channel 2;K03440 |
| 19 | K04914 | 1 | 0 | potassium channel subfamily K member 3;K05323 |
| 20 | K05323 | 2 | 0 | potassium channel subfamily K, invertebrate;K04924 |
| 21 | K05546 | 1 | 0 | alpha 1,3-glucosidase [EC:3.2.1.84] |
| 22 | K05695 | 1 | 0 | receptor-type tyrosine-protein phosphatase F [EC:3.1.3.48];K06792 |
| 23 | K05929 | 1 | 0 | phosphoethanolamine N-methyltransferase [EC:2.1.1.103] |
| 24 | K06002 | 1 | 0 | pepsin A [EC:3.4.23.1] |
| 25 | K06792 | 1 | 0 | aggrecan 1;K06793 |
| 26 | K07375 | 1 | 0 | tubulin beta |
| 27 | K07524 | 1 | 0 | regulator of G-protein signalling 3;K01062 |
| 28 | K07820 | 2 | 0 | beta-1,3-galactosyltransferase 2 [EC:2.4.1.-] |
| 29 | K08202 | 2 | 0 | MFS transporter, OCT family, solute carrier family 22 (organic cation transporter), member 4/5 |
| 30 | K08473 | 3 | 0 | nematode chemoreceptor |
| 31 | K08621 | 1 | 0 | a disintegrin and metalloproteinase with thrombospondin motifs 6 [EC:3.4.24.-] |
| 32 | K08635 | 1 | 0 | membrane metallo-endopeptidase-like 1 |
| 33 | K08726 | 1 | 0 | soluble epoxide hydrolase / lipid-phosphate phosphatase [EC:3.3.2.10 3.1.3.76];K11729 |
| 34 | K08765 | 1 | 0 | carnitine O-palmitoyltransferase 1, liver isoform [EC:2.3.1.21];K19523 |
| 35 | K09067 | 1 | 0 | achaete-scute complex protein |
| 36 | K09575 | 1 | 0 | FK506-binding protein 9/10 [EC:5.2.1.8] |
| 37 | K09614 | 1 | 0 | corin [EC:3.4.21.-];K10592 |
| 38 | K09886 | 1 | 0 | aquaglyceroporin related protein, invertebrate;K09877 |
| 39 | K10158 | 1 | 0 | galactosylgalactosylxylosylprotein 3-beta-glucuronosyltransferase 3 [EC:2.4.1.135];K00735 |
| 40 | K10956 | 1 | 0 | protein transport protein SEC61 subunit alpha |
| 41 | K11147 | 1 | 0 | dehydrogenase/reductase SDR family member 4 [EC:1.1.-.-] |
| 42 | K11154 | 1 | 0 | retinol dehydrogenase 16 [EC:1.1.1.-] |
| 43 | K11252 | 1 | 0 | histone H2B |
| 44 | K11989 | 1 | 0 | indian hedgehog |
| 45 | K12301 | 2 | SLC17A5 | MFS transporter, ACS family, solute carrier family 17 (sodium-dependent inorganic phosphate cotransporter), member 5;K08193 |
| 46 | K12307 | 5 | CLN7 | MFS transporter, ceroid-lipofuscinosis neuronal protein 7 |
| 47 | K12321 | 1 | 0 | guanylate cyclase 2D/E [EC:4.6.1.2] |
| 48 | K12354 | 1 | 0 | ectonucleotide pyrophosphatase/phosphodiesterase family member 7 [EC:3.1.4.12];K17601 |
| 49 | K12385 | 1 | NPC1 | Niemann-Pick C1 protein |
| 50 | K13208 | 1 | 0 | ELAV like protein 2/3/4 |
| 51 | K13862 | 1 | 0 | solute carrier family 4 (sodium borate transporter), member 11 |
| 52 | K14209 | 1 | 0 | solute carrier family 36 (proton-coupled amino acid transporter) |
| 53 | K14379 | 1 | ACP5 | tartrate-resistant acid phosphatase type 5 [EC:3.1.3.2] |
| 54 | K14410 | 1 | ACP2 | lysosomal acid phosphatase [EC:3.1.3.2];K01078 |
| 55 | K14453 | 1 | 0 | solute carrier family 26, other |
| 56 | K14620 | 1 | 0 | riboflavin transporter 2 |
| 57 | K14640 | 1 | 0 | solute carrier family 20 (sodium-dependent phosphate transporter);K03306 |
| 58 | K14943 | 1 | 0 | muscleblind |
| 59 | K15015 | 1 | 0 | solute carrier family 32 (vesicular inhibitory amino acid transporter);K14209 |
| 60 | K15734 | 1 | 0 | all-trans-retinol dehydrogenase (NAD+) [EC:1.1.1.105] |
| 61 | K16449 | 1 | 0 | regulator of G-protein signaling |
| 62 | K17623 | 1 | 0 | pseudouridine-5'-monophosphatase [EC:3.1.3.-] |
| 63 | K18399 | 1 | 0 | valacyclovir hydrolase [EC:3.1.-.-];K01A2.5; |
| 64 | K19176 | 1 | 0 | fatty acid amide hydrolase 2 [EC:3.5.1.99] |
| 65 | K19491 | 1 | 0 | doublesex- and mab-3-related transcription factor 4/5;K19492 |
| 66 | K19601 | 1 | 0 | protein eyes shut;K02599 |

**Table S4 Anti-phytoalexin genes related to the Lysosome pathway**

| **KEGG** | **Enriched genes** | **Lysosome related** | **Name** |
| --- | --- | --- | --- |
| K01373 | 13 | Lysosome | cathepsin [EC:3.4.22.41] |
| K12307 | 5 | CLN7 | MFS transporter, ceroid-lipofuscinosis neuronal protein 7 |
| K01052 | 4 | LIPA | lysosomal acid lipase |
| K12301 | 3 | SLC17A5 | MFS transporter, ACS family, solute carrier family 17, member 5 |
| K14410 | 2 | ACP2 | lysosomal acid phosphatase [EC:3.1.3.2] |
| K01201 | 1 | GBA | glucosylceramidase [EC:3.2.1.45] |
| K01369 | 1 | LGMN | legumain [EC:3.4.22.34] |
| K12347 | 1 | NRAMP | natural resistance-associated macrophage protein |
| K12350 | 1 | SMPD1 | sphingomyelin phosphodiesterase [EC:3.1.4.12] |
| K12385 | 1 | NPC1 | Niemann-Pick C1 protein |
| K14379 | 1 | ACP5 | tartrate-resistant acid phosphatase type 5 [EC:3.1.3.2] |
| Total | 33 |  |  |

**Table S5 The number of genes in the 25 modules**

| Module colors | Gene number |
| --- | --- |
| black | 517 |
| blue | 4468 |
| brown | 1074 |
| cyan | 154 |
| darkgreen | 36 |
| darkgrey | 31 |
| darkred | 38 |
| darkturquoise | 34 |
| green | 740 |
| greenyellow | 347 |
| grey | 2 |
| grey60 | 96 |
| lightcyan | 97 |
| lightgreen | 56 |
| lightyellow | 53 |
| magenta | 376 |
| midnightblue | 139 |
| pink | 395 |
| purple | 364 |
| red | 723 |
| royalblue | 47 |
| salmon | 177 |
| tan | 271 |
| turquoise | 6493 |
| yellow | 896 |

**Table S6 *Bx-cathepsin W* correlated anti-phytoalexin genes**

| **No** | **Blast nr** | **Log_2_(inoculation/CK)** | | | | |
| --- | --- | --- | --- | --- | --- | --- |
|  |  | *Pm* 1d | *Pm* 15d | *Ps* 15d | *Pt* 1d | *Pt* 15d |
| 1 | malate dehydrogenase | 1.46 | 0.86 | 1.14 | 1.37 | 1.06 |
| 2 | hypothetical protein PRIPAC_282, partial [*Pristionchus pacificus*] | 2.19 | 1.43 | 1.35 | 2.24 | 1.57 |
| 3 | N/A | 3.10 | 2.19 | 2.55 | 3.37 | 2.24 |
| 4 | ELAV-like protein 4 [*Toxocara canis*] | 1.03 | 0.67 | 0.73 | 1.20 | 0.57 |
| 5 | Protein CBR-LIPL-1  [*Caenorhabditis briggsae*] | 4.03 | 2.86 | 2.00 | 4.73 | 3.63 |
| 6 | N/A | 3.48 | 2.75 | 2.20 | 3.38 | 1.89 |
| 7 | N/A | 3.94 | 1.48 | 1.20 | 3.71 | 1.69 |
| 8 | Helix-loop-helix DNA-binding domain protein [*Necator americanus*] | 3.78 | 2.13 | 1.61 | 2.97 | 2.44 |
| 9 | Sterol-sensing domain and Patched family-containing protein [*Strongyloides ratti*] | 1.58 | 0.66 | 0.72 | 1.22 | 1.20 |
| 10 | N/A | 3.92 | 1.78 | 1.77 | 3.64 | 2.04 |

**Table S7 Identification of *B. xylophilus* xenobiotic metabolism genes**

|  | **Reactions** | **Bx Genome [10]{Kikuchi, 2011 #3}{Kikuchi, 2011 #16}** | **Ce** | **Bx transcriptome** |
| --- | --- | --- | --- | --- |
| **Phase I** |  |  | **373** | **551** |
| Cytochrome P450 monooxygenases | Oxidation, reduction, peroxidation | **76** | **81** | **68** |
| Flavin-containing monooxygenases | Oxidation |  | **5** | **16** |
| Alcohol dehydrogenases | Alcohol oxidation |  | **30** | **117** |
| Aldehyde dehydrogenases | Aldehyde oxidation |  | **13** | **31** |
| Monoamine oxidase | Oxidative deamination |  | **0** | **0** |
| NADPH-cytochrome P450 reductase | Reduction |  | **43** | **34** |
| Carbonyl reductases | Reduction |  | **1** | **9** |
| Aldo-keto reductases | Reduction |  | **1** | **24** |
| NAD(P)H-quinone oxidoreductase | Quinone reduction |  | **1** | **12** |
| Epoxide hydrolases | Epoxide hydrolysis |  | **12** | **11** |
| Carboxylesterases | Hydrolysis of ester-containing xenobiotics |  | **2** | **56** |
| Deaminases | Hydrolytic deamination |  | **2** | **3** |
| **Phase II** |  |  |  |  |
| Glutathione S-transferases | Conjugation with glutathione | **67** | **44** | **59** |
| UDP glucuronosyltransferases | Conjugation with glucuronide | **60** | **65** | **68** |
| N-Acetyltransferases | Acetylation |  | **4** | **1** |
| Sulfotransferases | Conjugation with sulfate |  | **8** | **7** |
| **Phase III** |  |  |  |  |
| ATP-binding cassette transporter | Xenobiotic transport across cell membranes | **106** | **61** | **35** |
| Lung resistance-related protein homologous | Nucleocytoplasmic transport |  | **0** | **0** |

**Text S1 Transcriptome library preparation and sequencing**

Transcriptome libraries were prepared and sequenced by BGI-Shenzhen (Shenzhen, China) according to the BGISEQ-500 standard protocol [1, 2]. The mRNA was purified from total RNA using oligo(dT)-attached magnetic beads and fragmented into small pieces. The first-strand cDNA was generated using reverse transcriptase and random primers. After the second-strand cDNA was synthesized, the cDNA was subjected to end-repair, 3′ adenylation, and adaptor-ligated. The double stranded cDNA was purified and enriched with PCR. The PCR products were heat denatured and circularized by the splint oligo sequence. DNA nanoballs (DNBs) were generated with the single stranded DNA circle by rolling circle replication (RCR) to intensify the fluorescent signals during the sequencing. The pair-end reads of 100 bp were read through on the BGISEQ-500.

**Text S2 Data analysis of reads**

Low-quality reads and trimming adaptor sequences were removed to acquire clean reads using SOAPnuke (v1.5.2, https:github.com/BGI-flexlab/SOAPnuke). Clean reads were matched to reference sequences using Bowtie2 (v2.2.5, http://bowtie-bio.sourceforge.net/Bowtie2/index.shtml) [3]. After clean reads were obtained, HISAT was used to compare clean reads to the reference genome sequence [4]. After comparing with the reference genome, StringTie (v1.0.4, http://ccb.jhu.edu/software/stringtie) was used to reconstruct the transcript of each sample, and Cuffcompare (one of the tools of Cufflinks, v2.2.1, http://cole-trapnell-lab.github.io/cufflinks) was used to compare the reconstructed transcript with the reference annotation information to obtain the new transcript, and CPC (v0.9-r2, http://cpc.cbi.pku.edu.cn) software was used to predict the coding potential of the new transcript. Clean reads were matched to reference sequences using Bowtie 2.

**Text S3 Transcriptome assembly**

All clean reads of the samples were merged into one big pseudo sample and the merged clean reads were assembled using Trinity (http://trinityrnaseq.sourceforge.net) [5] with default parameters. The assembled contigs were mapped to the reference genome using BLAT (http://genome.ucsc.edu) [6] and GMAP (http://research-pub.gene.com/gmap/) [7]. The alignments were validated and combined and the original genome annotation was updated using PASA (Program to Assemble Spliced Alignments, https://pasapipeline.github.io/) [8].

**Text S4 The differential gene expression analysis**

Expression levels of genes were calculated using RSEM (RNA-Seq by Expectation Maximization, v1.2.12, http://deweylab.biostat.wisc.edu/RSEM) [9]. DESeq [10] Bioconductor package was applied to perform differentially expressed genes (DEGs) detection. The raw counts of each gene were normalized to FPKM (Fragments Per Kilobase per Million mapped fragments) to adjust for different sequencing depths across samples using DESeq. After estimating the dispersion of each gene, differentially expressed genes were identified by DESeq using adjusted false discovery rate (FDR) [11]. In this study, DEGs were defined by default as those with FDR≤0.05 and multiple differences of more than 2 times (log_2_ fold change>1 or <-1).

**Text S5 The WGCNA algorithm**

WGCNA algorithm was used to evaluate the gene expression value. Power value was screened out in the construction of module by WGCNA algorithm. The gradient method was used to test the independence and the average connectivity degree of different modules with different power value. The appropriate power value was determined when the degree of independence was over 0.8. Once the power value was determined, the module construction proceeded by WGCNA algorithm. The minimum number of genes was set as 30 for the high reliability of the results. The WGCNA algorithm is employed to identify the co-expression modules. Module-trait associations were estimated using the correlation between the module eigengene and the traits. For each expression profile, gene significance (GS) was calculated as the absolute value of the correlation between expression profile and each trait; module membership (MM) was defined as the correlation of expression profile and each module eigengene

**References**

1. Huang J, Liang X, Xuan Y, Geng C, Li Y, Lu H, Qu S, Mei X, Chen H, Yu T: **A reference human genome dataset of the BGISEQ-500 sequencer**. *Gigascience* 2017, **6**(5):1-9.

2. Zhu FY, Chen MX, Ye NH, Qiao WM, Gao B, Law WK, Tian Y, Zhang D, Zhang D, Liu TY: **Comparative performance of the BGISEQ-500 and Illumina HiSeq4000 sequencing platforms for transcriptome analysis in plants**. *Plant Methods* 2018, **14**(1):69.

3. Langmead B, Salzberg SL: **Fast gapped-read alignment with Bowtie 2**. *Nature methods* 2012, **9**(4):357.

4. Kikuchi T, Cotton JA, Dalzell JJ, Hasegawa K, Kanzaki N, McVeigh P, Takanashi T, Tsai IJ, Assefa SA, Cock PJA *et al*: **Genomic Insights into the Origin of Parasitism in the Emerging Plant Pathogen *Bursaphelenchus xylophilus***. *Plos Pathog* 2011, **7**(9).

5. Haas BJ, Papanicolaou A, Yassour M, Grabherr M, Blood PD, Bowden J, Couger MB, Eccles D, Li B, Lieber M: **De novo transcript sequence reconstruction from RNA-seq using the Trinity platform for reference generation and analysis**. *Nature protocols* 2013, **8**(8):1494-1512.

6. Kent WJ: **BLAT—the BLAST-like alignment tool**. *Genome research* 2002, **12**(4):656-664.

7. Wu TD, Watanabe CK: **GMAP: a genomic mapping and alignment program for mRNA and EST sequences**. *Bioinformatics* 2005, **21**(9):1859-1875.

8. Haas BJ, Delcher AL, Mount SM, Wortman JR, Smith Jr RK, Hannick LI, Maiti R, Ronning CM, Rusch DB, Town CD: **Improving the Arabidopsis genome annotation using maximal transcript alignment assemblies**. *Nucleic acids research* 2003, **31**(19):5654-5666.

9. Li B, Dewey CN: **RSEM: accurate transcript quantification from RNA-Seq data with or without a reference genome**. *BMC bioinformatics* 2011, **12**(1):323.

10. Anders S, Huber W: **Differential expression analysis for sequence count data**. *Genome biology* 2010, **11**(10):R106.

11. Benjamini Y, Yekutieli D: **The control of the false discovery rate in multiple testing under dependency**. *Annals of Statistics* 2001, **29**(4):1165-1188.
